# Teratoma occupying the left hemithorax

**DOI:** 10.1186/1477-7819-3-76

**Published:** 2005-11-22

**Authors:** Charalambos Zisis, Dimitra Rontogianni, Grigorios Stratakos, Konstantinos Voutetakis, Konstantinos Skevis, Mihalis Argiriou, Ion Bellenis

**Affiliations:** 1 Department of Cardiothoracic Surgery, Evangelismos General Hospital, Athens; 2 Department of Pathology, Evangelismos General Hospital, Athens; 3 Department of Critical Care and Respiratory disease, Evangelismos General Hospital, Ipsilantou 45-47, Athens

## Abstract

**Background:**

Teratomas are manifested with a great variety of clinical and radiological features, while sometimes they simply represent incidental findings.

**Case presentation:**

A rare case of benign teratoma of the dermoid cyst type, in an adult 40-year-old female patient, is reported. The patient had presented recurrent pulmonary infections for the previous 2 months, persistent cough, and progressively aggravating dyspnea. A chest X-ray showed total atelectasis of the left lung, and the thoracic CT-scan revealed a huge mass, containing multiple elements of heterogeneous density, probably originating from the mediastinum, occupying the whole left hemithorax. The mass compressed the vital structures of the mediastinum, great vessels and airways, and a chest MRI was performed to accurately detect the anatomical relations. The patient underwent left thoracotomy and the tumor was totally resected. The size of the tumor was extremely large although no invasion to the vessels or to the airway had occurred. Adherence to the adjacent left pulmonary artery and left main bronchus was present, but without erosion or fistulization. The postoperative course was uneventful, while the histological examination confirmed a teratoma.

**Conclusion:**

A teratoma is a non-homogeneous pathological entity, clinically, radiologically or histologically. It is predominantly diagnosed between the second and fourth decade and the incidence is equal for both sexes. Symptoms are absent in one half of the patients. The case reported is noteworthy as the tumor appeared with total atelectasis of the left lung, and symptoms started 2 months prior to diagnosis. Total removal of the tumor is adequate treatment for this type of teratoma and the prognosis is excellent.

## Background

Mature teratomas are the most common histological type of germ cell tumors, followed by seminomas [1]. Germ cell tumors are predominantly found in gonads, while the anterior mediastinum is the most common extragonadal site [2]. The mediastinal germ cell tumors comprise 15% of anterior mediastinal tumors in adults and 25% in children [3]. According to the mediastinal germ cell tumor classification system proposed in 1986 by Mullen and Richardson, there are three categories: benign germ cell tumors, seminomas, and nonseminomatous germ cell tumors, also called malignant teratomas [4]. The benign germ cell tumors are also called epidermoid cysts, benign teratomas, or simply teratomas. These tumors can characteristically be cystic or solid or a combination of the two, contain multiple germ cell layers (sometimes all three are recognized, i.e., ectoderm, mesoderm, and endoderm), and are composed of tissue foreign to the organ or anatomic site in which they arise.

## Case presentation

**Figure 1:**
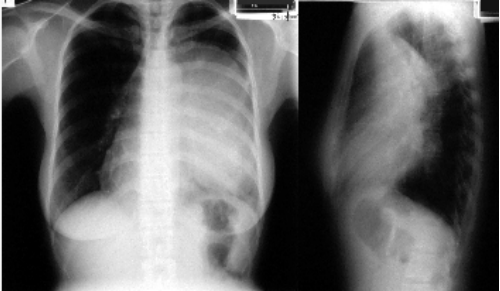
Preoperative chest X-ray showing total atelectasis of the left lung and deviation of the cardiac silhouette to the right.

**Figure 2:**
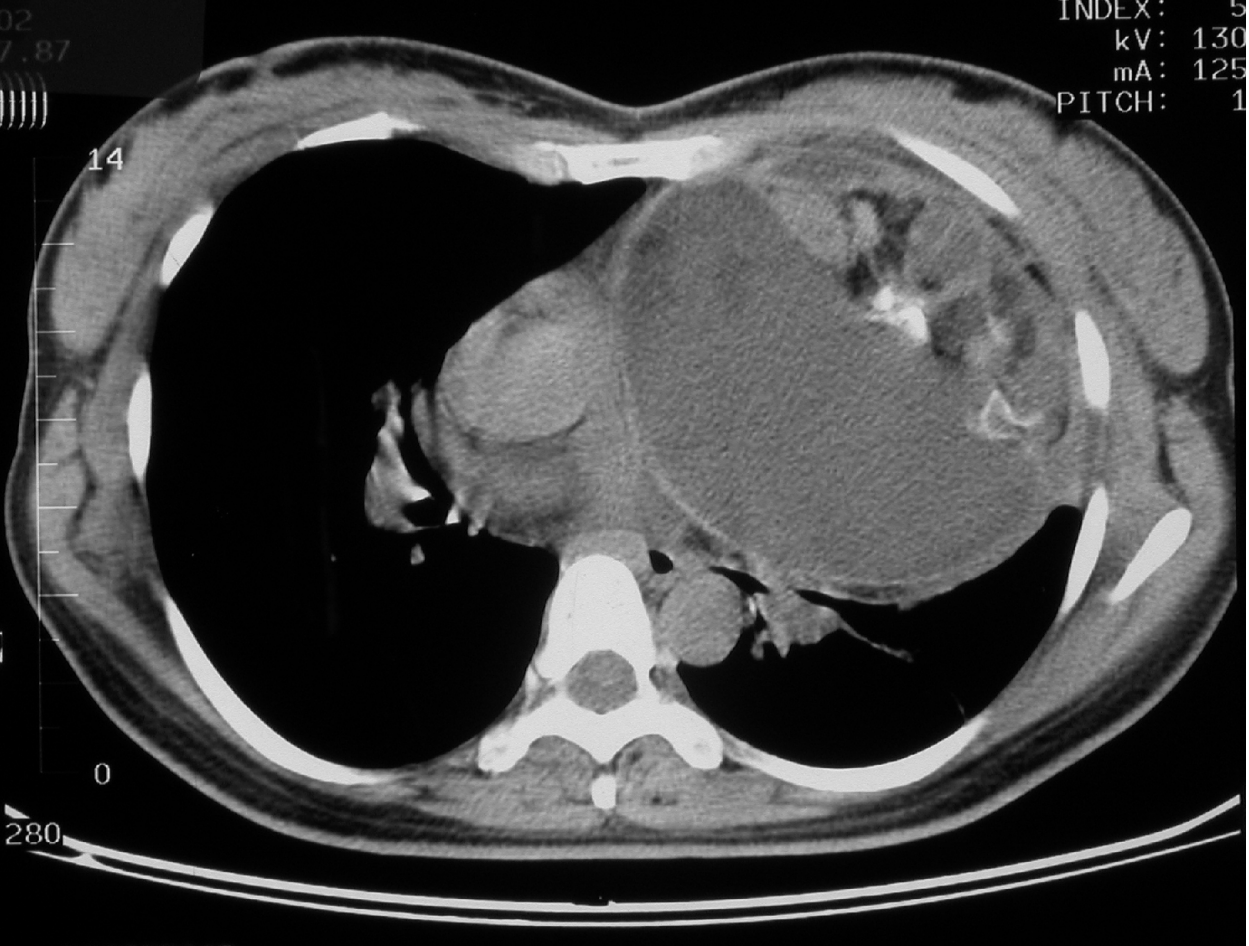
Preoperative CT-scan of the chest revealing the mass compressing the mediastinal vessels and airway.

**Figure 3:**
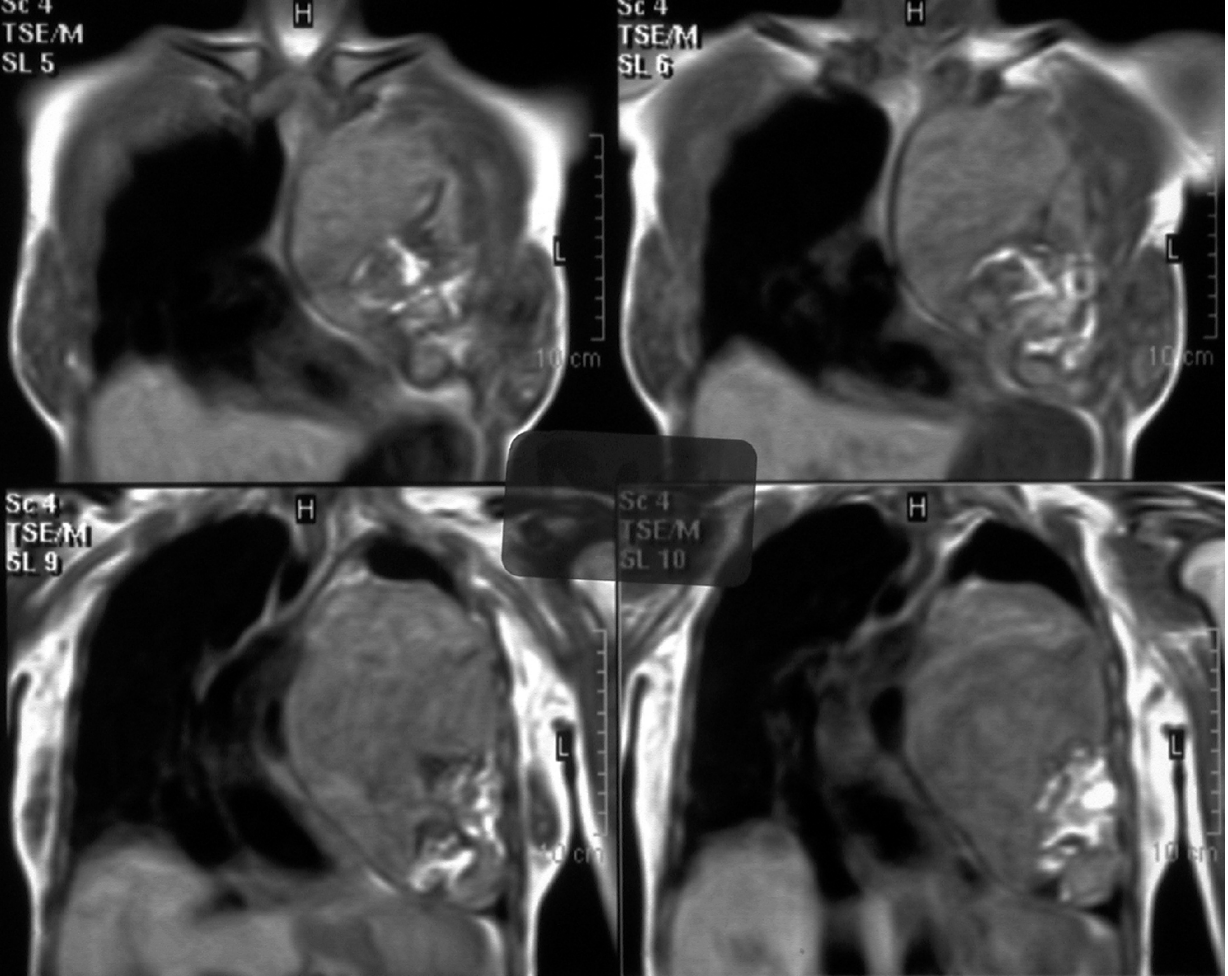
Preoperative MRI showing the teratoma, its anatomic relations to the mediastinum, and its expansion to the left hemithorax.

A 40-year-old female with negative smoking and medical history was admitted with productive cough, progressively aggravating dyspnea on exertion, and recurrent pulmonary infections for the previous 2 months. The chest X-ray showed total atelectasis of the left lung (Figure 1), and the thoracic CT-scan revealed a mass of the left hemithorax, which probably originated in the mediastinum and extended to the whole left pleural space (Figure 2). The mass showed heterogeneous density containing soft tissue elements, fat, cystic areas and foci of calcification, which is the classic imaging appearance of a benign teratoma on CT. Magnetic resonance imaging (MRI) was performed to specify the anatomic relationships and confirm the tumor morphologic features. The MRI yielded useful information about the vital structures of the mediastinum, whether invaded or externally compressed by the tumor. Specifically, the MRI confirmed a round, non-homogeneous, well circumscribed mass of a 12 cm diameter, exerting compression on the mediastinum great vessels and the left hilar structures (vessels and airway) (Figure 3). The bronchoscopy found stenosis of the trachea by external compression, and narrowing in the foramen of the left main bronchus resulting in difficulty in the insertion of the bronchoscope. The mediastinal tumor markers (α-fetoprotein and β-human chorionic gonadotropin) were both normal. As the findings of CT and MRI suggested a benign teratoma, a complete resection was contemplated. Since the mass was supposed to be respectable, surgical management was first in the priority list of therapeutic options. The cytological examination through transcutaneous needle aspiration or biopsy of the tumor were considered redundant and were omitted, because of the dispersion risk and the necessity for total removal so as to ameliorate the respiratory function and re-expand the left lung. Moreover, needle biopsy allows examination of only a small amount of tissue and may be inadequate for definitive diagnosis [5]. As already underlined in the literature, diagnosis and therapy rely on surgical excision, and even with large sized tumors whose complete resection is impossible, partial resection still relieves symptoms, frequently without relapse [6]. Pulmonary function tests were impaired: FVC 1.19 (36.7% of predicted) and FEV1 1.01 (41.5% of predicted), whereas a-FP, βCG were normal.

**Figure 4:**
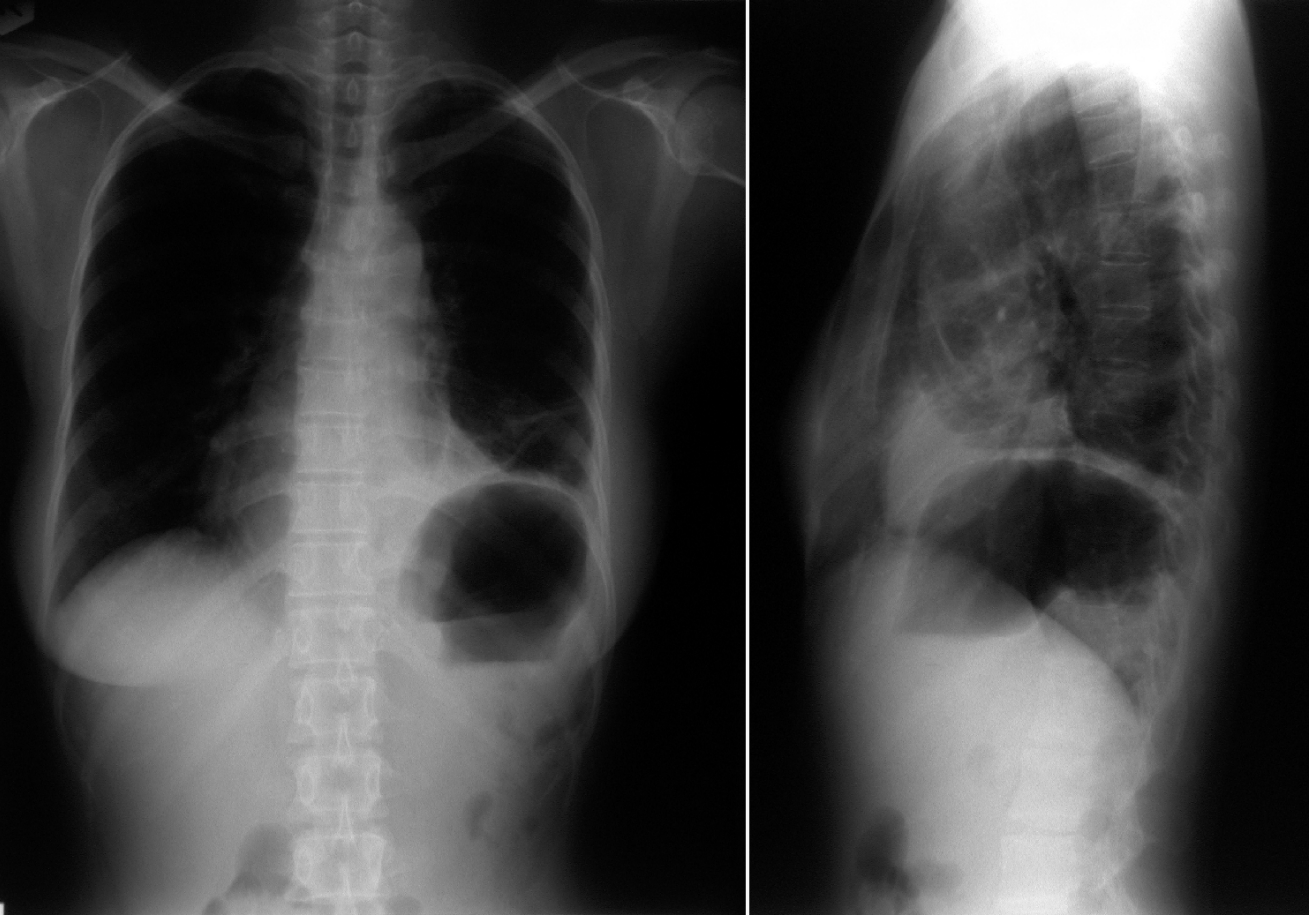
Postoperative X-ray of the chest after total resection of the teratoma, showing re-expansion of the left lung.

The patient underwent a total resection of the mediastinal mass via a left posterolateral thoracotomy. Entry into the pleural space was performed through the fifth intercostal space, and, because of the tumor large size, extension of the incision was necessary to obtain safe visualization of the cavity and proceed to tumor mobilization. Approaching via left thoracotomy makes access to the mediastinal structures difficult but permits control of the whole hemithorax up to the hilar structures. Many adhesions existed with the left pulmonary artery, the left main bronchus, the pericardium, the aorta, and the diaphragm, and a combination of blunt and sharp dissection for the division was applied uneventfully. Because of difficulty in the mobilization of such a huge mass, a purse string suture permitted aspiration of sebaceous content via a small incision in the wall. As the size diminished, manipulation was facilitated. The tumor, excised en block, was white-gray colored, well circumscribed, and thick capsuled. A tube thoracostomy was introduced, the collapsed left lung was easily re-expanded, and the patient was extubated. The patient recovered well from the operation and was discharged on the 2^nd ^postoperative day. Preoperative atelectasis of the left lung was totally resolved (Figure 4), and the pathological examination revealed a benign mature teratoma with dermoid cyst characteristics, containing sebaceous and gelatinous material. Two years later, the patient is doing well out of recurrence.

## Discussion

Germ cell tumors typically occur in young adults in their second to fourth decade with equal sex distribution. Female predominance has been reported by some authors with a 1.27–2.05: 1 female: male ratio [7-9]. Multicompartment extension is observed in 10–15% of the cases [2]. According to another review, 3–8% are located in the posterior portion of the visceral compartment or the paravertebral regions [10,11], where neurogenic tumors and neoplastic lymphadenopathy commonly exist. In the case reported, the teratoma occupied at least two of the three mediastinal compartments (the anterior and the middle) exerting compression on relevant vital anatomical structures.

Nearly one half of patients (36–62% in various series) have no signs or symptoms when the mass is initially diagnosed. Symptoms commonly present are chest, back or shoulder pain, dyspnea, cough, fever, pleural effusion, and bulging of the chest wall [7]. In the case under discussion, the patient presented symptoms from her tumor for 2 months, either due to tumor inflammation or the total atelectasis of the lung, following recurrent pulmonary infections. Such a sizeable tumor was thus asymptomatic for a long period. Hemoptysis or expectoration of hair or sebum can rarely occur when communication between the tumor and the tracheobronchial tree develops. Symptoms can also derive from the pressure exerted on the surrounding tissues (for example superior vena cava syndrome).

In spite of the pressure on the mediastinal vessels or the airway, invasion of these anatomical elements does not usually occur. However, erosion of the tumor to the bronchus, as well as to the pleural space, to the skin, and to the aorta has been reported.

Diagnostic assessment is performed with classical X-rays, followed-up by CT. Typically, a well-circumscribed anterior mediastinal mass extending to one side of the midline and protruding into one lung field is revealed. CT accurately estimates the density of all included tissues, such as soft tissue (in virtually all cases), fluid (88%), fat (76%), calcification (53%), and teeth and such imaging findings are considered specific [2]. MRI is valuable in detecting the anatomic relations to the mediastinal and the hilar structures, such as vessels and airways [12].

Macroscopically the tumors are spherical, lobulated, with a well-defined capsule, and contain a variety of material, lipid-rich fluid, cheese-like substances, teeth, hair, and cartilage. Histologically, benign teratomas comprise at least 2 of the 3 primordial layers: ectoderm (skin and hair), mesoderm (bone, fat, and muscle), and endoderm (respiratory epithelium and gastrointestinal tract).

Surgical resection is the treatment of choice and radical extirpation secures a long survival out of recurrence. Median sternotomy is usually preferred for tumor removal, but access via either posterolateral or anteroposterior thoracotomy depends on the size, location, and expansion of the tumor. Difficulty in surgical maneuvers may be a result of the vital structures involved. In a large series of 95 patients with benign mature teratoma, in addition to tumor resection, 3 patients required lobectomy, 5 patients additional partial resection of the lung, and 7 patients pericardectomy. VATS techniques have been introduced in teratoma resection with promising results [7,13]. The appropriate operative procedure choice, depending on the patient's age, tumor size, location, expansion, psychological profile, coexistent morbidity, cardiorespiratory reserve, and preference, as well as the surgeon's experience guarantee a favorable outcome and long-term prognosis.

In the case reported, left posterolateral access was chosen, as the tumor was almost entirely located into the left hemithorax, reaching and adherent to the left hemidiaphragm. Such a location precludes median sternotomy, otherwise the preferred approach, as surgical manipulations are impossible on the lower lobe of the left lung and on the left hemidiaphragm. MRI was performed after the CT-scan in order to more precisely reveal the relations with the mediastinum vital anatomical structures (major vessels, aorta, pulmonary artery) because it was critical to clarify whether there was an invasion or simply compression. The examination pointed to the latter and loose tumor adhesions with the great vessels were confirmed on surgery and gently submitted to blunt dissection. The left lateral decubitus position of the patient did not influence ventilation during operation, as ventilation conditions with a double lumen tracheal tube were similar to the preoperative values affected by the left lung atelectacis. Furthermore, left lung operation secured the right lung against potential contamination from infected content of the chronically collapsed left lung.

## Conclusion

• Such a huge teratoma extending to the whole hemithorax and resulting in total atelectasis of the left lung has been only sparsely reported previously [14,15].

• Even a huge mass causes simple compression and deviation without invasion of the vascular and airway structures.

• Complete resection is adequate treatment for the patient with favorable long-term prognosis.

## Conflict of interest

The author(s) declare that they have no competing interests.

## Authors' contributions

**CZ **was the main surgeon of the patient, who reviewed the literature and had the central responsibilityin the management ofthe patient and the writing of this report,

**DR **was the pathologist, who examined and diagnosed histologically the resected specimen,

**GS **was the interventional bronchoscopist and pulmonologist, who performed bronchoscopy, functional respiratory tests and assessed the patient as candidate of major surgical procedure,

**KV **and **KS **were assistants in this operation and helped in preparation of the manuscript,

**MA **offered his valuable experience in the management of the patient and the writing of this report,

**IB **coordinated writing of the manuscript.

All authors read and approved the final manuscript.
